# Heterostructure engineering for efficient halide perovskite X-ray detectors

**DOI:** 10.1016/j.isci.2026.117442

**Published:** 2026-09-05

**Authors:** Feng Li

**Affiliations:** 1 School of Physics, The University of Sydney, Sydney, NSW 2006, Australia

**Keywords:** perovskite, X-ray detector, heterostructure, interface, imaging

## Abstract

Halide perovskites have demonstrated great potential for next-generation X-ray detectors owing to their strong X-ray absorption, excellent charge-transport properties, tunable band structures, and low-cost processability. These advantages enable high-sensitivity, low-dose, and fast-response X-ray detection for medical imaging, security screening, and industrial inspection. Recently, heterostructure engineering has become an effective strategy for improving detector performance and stability by regulating band alignment, interfacial coupling, and carrier transport. In this review, we highlight recent advances in heterostructure-engineered halide perovskite X-ray detectors, including perovskite/perovskite and perovskite/functional-material heterostructures. Particular emphasis is placed on how heterointerfaces improve charge separation and transport, suppress recombination losses, inhibit ion migration, and enhance device stability and imaging performance. Finally, we discuss the remaining challenges and future opportunities for developing high-performance, stable, and scalable perovskite X-ray detection technologies.

## Graphical abstract

**Figure:**
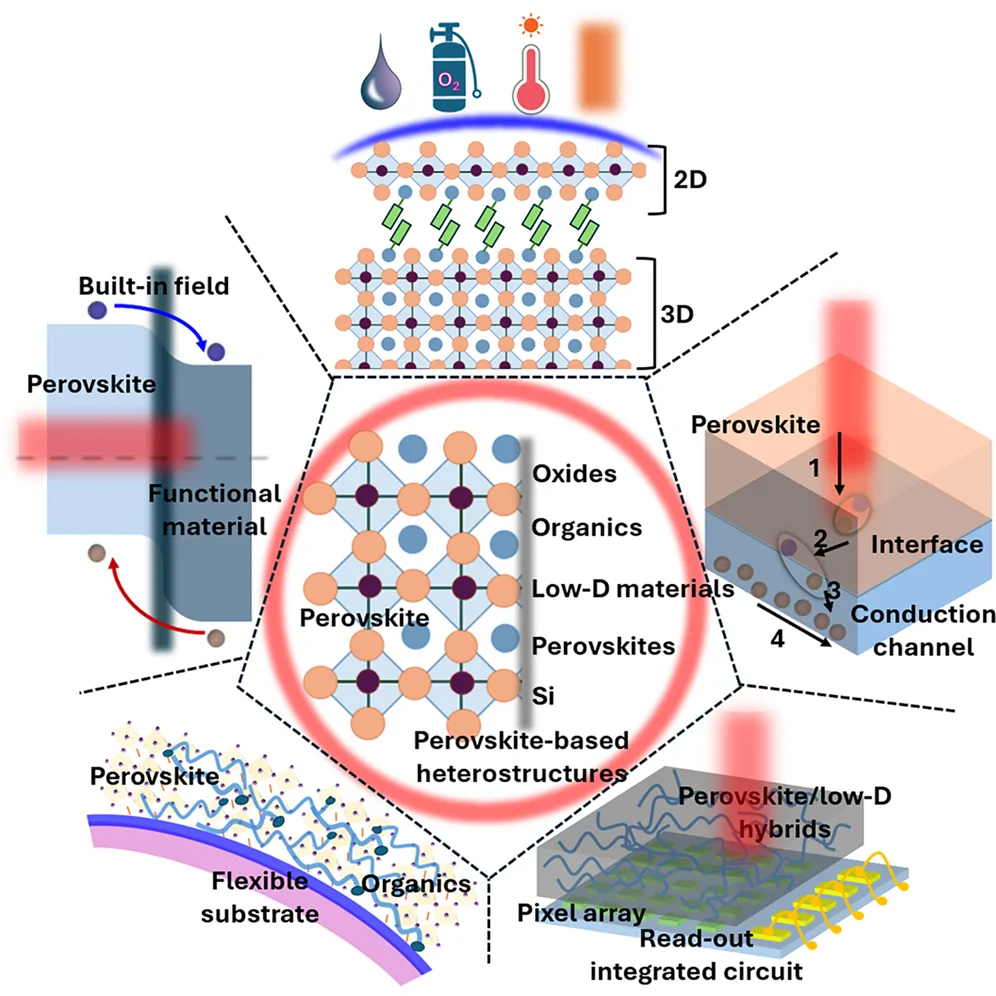

## Teaser

Applied sciences; Materials characterization techniques; Materials physics

## Introduction

X-ray detection plays a central role in the application fields, including medical diagnostics, non-destructive industrial radiography, scientific imaging research, and security inspection.^1^^,^^2^^,^^3^^,^^4^ Since the discovery of X-rays by W.C. Röntgen in 1895, X-ray imaging was established as the first medical imaging technique and has continuously evolved from conventional radiography to advanced modalities such as computed tomography (CT), angiography, and mammography, while also enabling major advances in industrial inspection, scientific research, and astronomical observations.^1^^,^^5^^,^^6^^,^^7^ Nowadays, state-of-the-art commercial X-ray imaging systems mainly rely on flat-panel X-ray detectors, which, depending on the X-ray conversion mechanisms, can normally operate through the indirect-conversion and direct-conversion mechanisms.^1^^,^^8^

Indirect-model X-ray detectors employ scintillators to convert high-energy X-rays to low-energy visible or UV light, which is subsequently detected by the extra photodetectors or charge-coupled devices (CCDs).^1^^,^^8^ Traditional scintillator materials, mainly including alkali metal halides (CsI and NaI), bismuth germanate (Bi_4_Ge_3_O_12_), and gadolinium oxysulfide (Gd_2_O_2_S),^1^^,^^9^^,^^10^ have enabled mature imaging technologies but often require complex and costly fabrication processes and suffer from limitations such as slow scintillation decay, limited flexibility, suboptimal light yield (LY), and poor mechanical properties that are not capable with the flexible systems.^10^ Direct-model X-ray detectors, in contrast, directly convert X-ray photons into electrical signals, offering simplified architectures and improved spatial resolution.^1^^,^^8^^,^^10^ Commercial direct-conversion materials such as high-purity Si, stabilized *α*-Se, cadmium zinc telluride (CdZnTe), mercuric iodide (HgI_2_), and lead oxide (PbO) have also demonstrated excellent detection performance.^10^^,^^11^^,^^12^ Notably, these materials for both indirect- and direct-model X-ray detectors have gained considerable achievements, yet challenges including high fabrication cost, large dark current and noise level, material rigidity, and demanding processing conditions still hinder their widespread deployment, particularly in next-generation low-dose, cost-effective, and flexible imaging systems.^1^^,^^10^^,^^12^

Recently, halide perovskites have emerged as highly promising candidates for both indirect and direct X-ray detection devices owing to their strong X-ray attenuation coefficients, high carrier mobility-lifetime products, tunable electronic structures, defect tolerance, and low-temperature solution processability.^1^^,^^10^^,^^12^^,^^13^^,^^14^^,^^15^^,^^16^^,^^17^ Halide perovskites in the forms of single crystals, polycrystalline films, and nanostructures have demonstrated remarkable sensitivity and ultralow detection limits, even outperforming several conventional detector materials.^10^^,^^12^^,^^17^ More significantly, recent advances suggest that heterostructure engineering provides powerful opportunities to further optimize charge generation, separation, transport, and interfacial stability in halide perovskite X-ray detectors. By integrating different perovskite compositions or functional materials, the formed heterostructures can create favorable band alignments and built-in electric fields that enhance charge separation, transport, and collection while suppressing recombination and dark current. These architectures can also mitigate ion migration, improve operational stability, and enable higher sensitivity, faster response, and lower detection limits. Furthermore, combining perovskites with organic, low-dimensional, or wide-bandgap materials broadens device functionality and flexibility. Consequently, heterostructure engineering has emerged as a promising strategy for developing high-performance, stable, and scalable perovskite X-ray detectors.

In this review, we discuss recent progress in heterostructure engineering for efficient halide perovskite X-ray detectors. We first introduce the fundamentals and application requirements of X-ray detection, followed by the unique advantages of halide perovskites for X-ray sensing. Thereafter, we focus on heterostructure design strategies, including perovskite-perovskite and perovskite-other material heterojunctions, as well as interfacial engineering with electrodes and charge transport layers, toward low-cost, high-performance X-ray detectors. Finally, we highlight the remaining challenges and future opportunities for developing high-performance, stable, and scalable perovskite X-ray detection technologies.

## Fundamentals and applications of X-ray detectors

### Fundamentals of X-rays and X-ray/matter interaction

**Figure 1:**
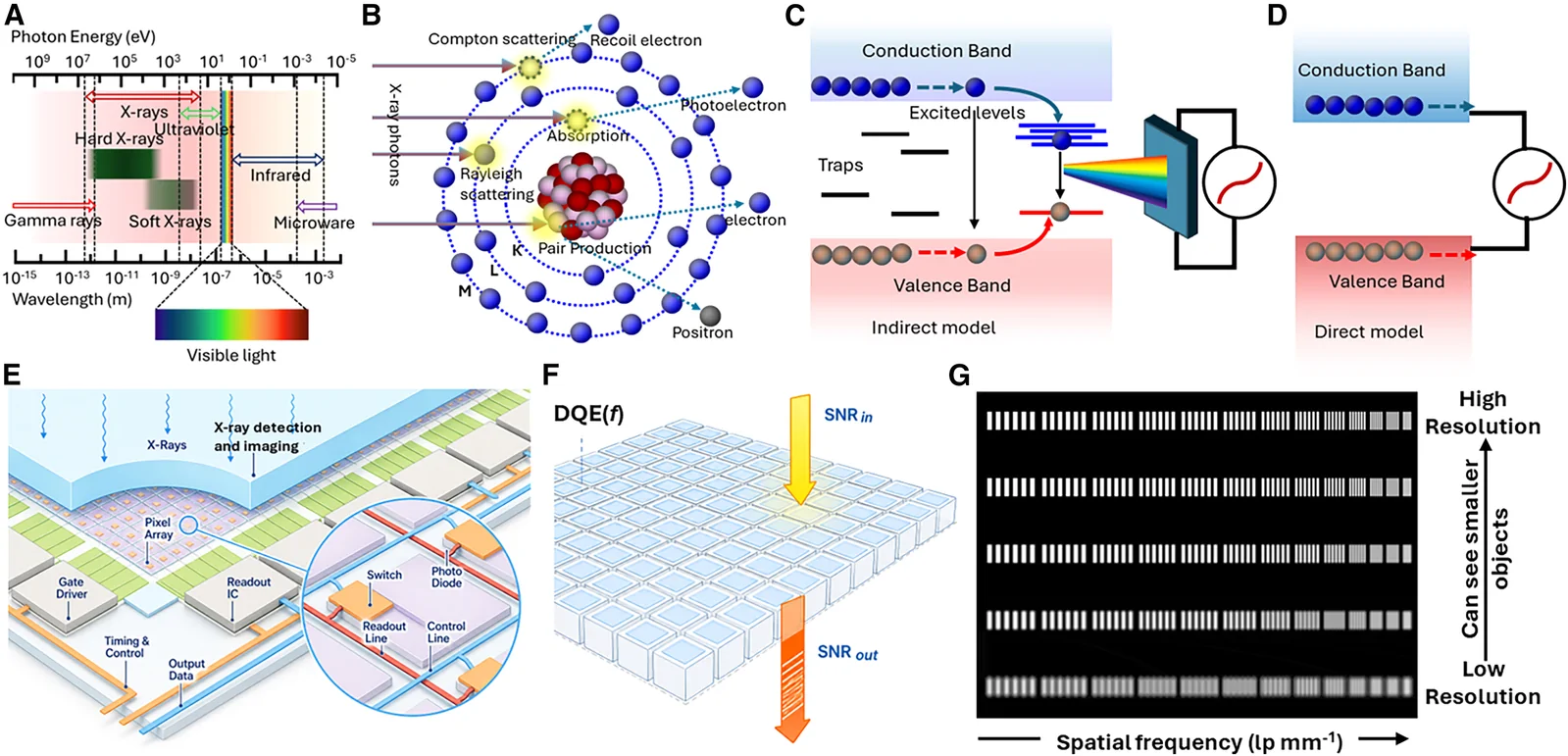
Fundamentals of X-rays and processes of X-ray detection and X-ray imaging (A) X-ray classification based on its wavelength and energy. The range of the visible (vis) wavelengths is magnified for clarity.^5^ (B) Interactions between X-ray photons and target materials: photoelectric absorption, Compton scattering, Rayleigh scattering, and electron-positron pair production. (C) The detection mechanism of indirect-type X-ray detector.^5^ (D) The detection mechanism of direct-type X-ray detector.^5^ (E) The schematic illustration of the internal construction of a flat-panel X-ray detector.^1^ (F) The schematic diagram of detective quantum efficiency. (G) The direct method for measuring the modulation transfer function (MTF) of X-ray imaging systems, aiming to determine the spatial resolution values. Reproduced with permission (A and C–E).

X-rays are high-energy electromagnetic waves with wavelengths ranging from approximately 10 nm to 10 p.m. and photon energies from approximately 0.1–100 keV, positioned between ultraviolet light and gamma rays in the electromagnetic spectrum (Figure 1A).^5^ Depending on their energy, X-rays are generally classified as “soft” X-rays (<10 keV) or “hard” X-rays (>10 keV).^5^^,^^8^^,^^18^ Owing to their strong penetration capability, hard X-rays are widely used in medical radiography, CT, industrial inspection, and security screening, whereas soft X-rays are commonly employed in microscopy and diffraction studies.^19^^,^^20^^,^^21^

The operation of X-ray detectors relies on the interaction between X-ray photons and absorbing materials. The dominant interaction mechanisms include photoelectric effect, Rayleigh scattering, Compton scattering, and electron-positron pair production (Figure 1B). For the energy range relevant to most imaging applications, the photoelectric effect dominates and is therefore particularly important for X-ray detection.^5^^,^^8^^,^^22^ In this process, X-ray photons are absorbed and generate energetic electrons, which subsequently produce cascaded ionization and electron-hole pairs within the material.

The efficiency of X-ray absorption is determined by the attenuation coefficient, which is a measure of how much the incident X-ray beam is weakened (either by absorption or scattering events) by the absorbing material that is passing through.^5^^,^^8^^,^^10^ Such a coefficient strongly depends on photon energy, material density and thickness, and atomic number (*Z*).^1^^,^^5^^,^^12^ Materials containing high-*Z* elements generally exhibit strong X-ray attenuation and therefore show great potential for X-ray detector application.^5^^,^^8^^,^^10^^,^^12^ The generated charge carriers can either recombine to produce low-energy photons in indirect detectors (Figure 1C) or be directly collected as electrical signals in direct detectors (Figure 1D).^5^

### Processes of X-ray detection

#### Indirect X-ray detection

Indirect X-ray detectors operate by converting high-energy X-ray photons into low-energy visible or UV photons through scintillators, followed by signal collection using photodetectors or CCDs (Figure 1C).^5^^,^^8^^,^^10^^,^^17^ The scintillation process generally involves three sequential steps: conversion, transport, and emission.^23^ Upon X-ray irradiation, energetic photons interact with the absorbing material and generate large numbers of electrons and holes through ionization processes. These carriers subsequently migrate through the material and recombine at luminescent centers to emit visible photons.^23^ Notably, the efficiency of scintillation is strongly influenced by carrier transport and recombination dynamics. During carrier migration, defects such as vacancies, grain boundaries, and impurities can trap charge carriers, resulting in nonradiative recombination, delayed emission, and energy loss.^5^^,^^10^ Therefore, improving crystallinity, reducing trap densities, and optimizing interfacial quality are critical for achieving efficient scintillation. Indirect X-ray detectors or scintillators remain widely used because of their low manufacturing cost, mature integration technology, fast response, and excellent operational stability.

Several figures-of-merit are commonly used to evaluate scintillator performance, which mainly include afterglow, LY, and emission wavelength.

The afterglow is defined as the time taken from the absorption of high-energy X-ray photons to the emission of UV or visible optical photons with low energy, which characterizes the residual luminescence after X-ray excitation is removed. It is estimated by measuring the decay time of the scintillation optical emission signal upon excitation. Excessive afterglow can lead to image lag and motion artifacts, particularly in high-speed imaging applications such as CT. Since the signal is superimposed on the previous exposure, long afterglow will cause imaging artifacts.^5^^,^^24^

LY, defined as the number of emitted photons per unit absorbed X-ray energy, determines the signal intensity and directly affects imaging sensitivity. Its value can be determined as: LY = 10^6^(*SQ*)/*βE*_g_, where *S* is the efficiency of transport of electron-hole (*e*-*h*) pairs, *Q* is the luminescence efficiency, and *E*_g_ is the energy bandgap, while *β* stands for the average energy required to generate a thermalized electron-hole pair.^5^^,^^23^^,^^24^ LY is governed by carrier transport efficiency, radiative recombination probability, and defect-assisted nonradiative losses. In particular, the recombination centers in the indirect-type X-ray detector or scintillator could induce radiative but also nonradiative recombination (non-emissive and hence a lossy process) due to the presence of defects and/or impurities, which further decrease the LY values. In the practical applications, a reflective coating is used to avoid light loss, and some optical coupling glue may be applied between the scintillators and the integrated photodiodes or CCDs to guarantee the light extraction efficiency.

In addition, the emission wavelength is another key parameter of the indirect X-ray detectors or scintillators. Emission is the visible or near-visible light emitted from the indirect X-ray detector or scintillators when excited by X-ray radiation. If the central wavelength of emission coincides with that of photoluminescence, it means that the two emitted lights come from the same energy level. Generally, the emission intensity boosts linearly with the enhancement of dose rate. The emission wavelength of the indirect X-ray detector or scintillator also needs to be optimized to improve the LY values. The emission wavelength of an indirect X-ray detector or scintillator should match the spectral response range of the integrated photodetector to maximize signal collection efficiency.^5^^,^^25^

#### Direct X-ray detection

Direct X-ray detectors convert the absorbed X-ray photons directly into electrical signals (Figure 1D). In this process, X-ray photons generate electron-hole pairs within the semiconductor absorber through photoelectric and scattering interactions. Under an applied electric field, the charge carriers drift toward opposite electrodes and are collected as photocurrent signals. Compared with indirect detectors, direct detection offers simplified device architectures, reduced signal loss, and improved spatial resolution.^6^ Direct X-ray detection can operate in either current mode or photon-counting mode, depending on the incident photon flux.^6^^,^^25^^,^^26^ Current-mode detectors are more commonly used for imaging applications because they provide continuous signal output with high signal-to-noise ratios (SNRs) and smooth image acquisition.^26^^,^^27^ In contrast, photon-counting detectors are suitable for low-flux applications and can provide enhanced energy discrimination.^28^

The performance of direct X-ray detectors is mainly evaluated using several key parameters. Although some particular applications may require different characteristics, the main figures-of-merit for performance evaluation include dark current, sensitivity, detection limit, and response speed.

##### Dark current

The dark current, also known as the leakage current, is one of the most critical factors because excessive dark current increases electronic noise, reduces dynamic range, and deteriorates SNR.^1^^,^^5^^,^^10^^,^^12^^,^^17^ Suppressing dark current typically requires high-resistivity materials, reduced defect densities, and optimized electrode interfaces, such as Schottky contacts that block unwanted carrier injection.

##### Sensitivity

Sensitivity (*S*) is a critical parameter to evaluate a direct-type X-ray detector, which describes the electrical response generated per unit X-ray dose and reflects the efficiency of charge generation, separation, and collection.^1^^,^^10^^,^^12^^,^^17^ High sensitivity indicates that X-ray can generate a high photocurrent under the radiation at a given dose rate, and it is highly related to the imaging quality. Its value can be determined by the collected charge per unit area under X-ray radiation: S = (*I*_X_ – *I*_D_)/(*D* × *V*), where *I*_X_ and *I*_D_ are the current values upon X-ray radiation and in the dark, respectively, *D* is the X-ray dose rate, and *V* is the effective detection area or volume.^1^^,^^10^^,^^12^^,^^17^ According to this formula, one effective way to improve the sensitivity value is to increase the applied electric field, which can facilitate separation of X-ray-induced charge carriers and reduce recombination (increasing *I*_X_). However, a high electric field always causes a large dark current and thus affects the performance of the X-ray detector. Thus, a suitable electric field is necessary to achieve high X-ray detection sensitivity. Currently, high sensitivity is generally achieved through strong X-ray absorption, efficient carrier transport, and minimized recombination losses.

##### Charge-collection efficiency

Closely related to sensitivity is the charge-collection efficiency (CCE),^1^^,^^5^ which represents the fraction of generated carriers successfully extracted by the electrodes. Its value can be calculated from the X-ray-induced current (*I*_X_) and theoretical radiation current (*I*_R_) with the following equation: CCE = *I*_X_/*I*_R_.^1^^,^^5^ There are normally two approaches for enhancing the CCE values. The first one is to avoid charge trapping while increasing the drift length of charge carriers by utilizing a high bias. The second one is to improve the quality of the active materials (e.g., reducing the trap densities and defects).^5^

##### Detection limit

The detection limit quantifies the minimum detectable X-ray dose rate that can be reliably recognized by an X-ray detector and is defined as the equivalent dose rate that produces a response signal 3 times larger than the noise level (SNR of 3), according to the International Union of Pure and Applied Chemistry (IUPAC).^1^^,^^5^^,^^28^ The detection limit is determined by both signal sensitivity and system noise.^29^^,^^30^ The key to achieving a relatively low detection limit is to obtain a high current signal at a low noise level. Besides, low dark current is also an important prerequisite to achieve low detection limit, because under the same rate of X-ray radiation, the lower dark current is conducive to the X-ray detector to obtain the higher SNR. In addition to the dark current, low ion mobility in active materials would also help the related detector achieve a stable X-ray response, further improving the SNR and lowering the detection limit. Low detection limits are particularly important for medical imaging because they enable reduced radiation exposure.^1^^,^^29^^,^^30^

##### Carrier mobility-lifetime product

Another important parameter is the carrier mobility-lifetime (*μτ*) product, which reflects the average drift distance of carriers before recombination. Its value for the particular active materials can be derived by fitting the X-ray radiation-induced current (*I*) curve using the modified Hecht formula^5^^,^^8^^,^^10^^,^^12^: *I* = (*I*_0_*μτV*/*L*^2^)[(1 – exp(-*L*^2^/*μτV*))/(1 + *Ls*/*Vμ*)], where *I*_0_ is the saturated X-ray-induced current, *L* is the thickness of active layer, *V* is the applied bias, and *s* is the surface recombination velocity. A large *μτ* product generally indicates efficient carrier transport and high detector performance.

##### Response speed

Response speed determines how rapidly a detector responds to X-ray irradiation and is essential for dynamic imaging and real-time monitoring.^1^^,^^5^^,^^8^^,^^29^ Similar to the general photodetectors working in UV and/or visible light range, the response speed of direct X-ray detectors is quantitatively defined as the rise time of the pulse peak from 10% to 90% (rise time, *τ*_1_) and then from 90% to 10% (decay time, *τ*_2_) along with the required stable output time. Slow response is often associated with carrier trapping and ion migration; therefore, developing high-quality materials and well-engineered interfaces is critical for achieving fast and stable X-ray detection.^31^

These considerations are especially relevant for halide perovskite detectors, where heterostructure engineering has emerged as a powerful strategy for simultaneously optimizing charge transport, interfacial recombination, and operational stability.

### X-ray detection and imaging applications

#### X-ray detection/imaging system

The X-ray imaging relies on the different attenuation capabilities of materials toward incident X-ray photons, generating contrast that reveals the internal structures and compositions of objects. In a typical X-ray imaging system, transmitted X-ray photons are recorded by detectors operating in either indirect or direct conversion modes, and the resulting electrical or optical signals are reconstructed into two-dimensional radiographic or three-dimensional tomographic images. Modern flat-panel X-ray detectors dominate current imaging technologies because of their rapid readout, large-area integration, high spatial resolution, and compatibility with digital image processing (Figure 1E).

Since the discovery of X-rays by W. Röntgen in 1890, X-ray imaging has evolved into one of the most important non-invasive diagnostic and analytical tools across medicine, industry, scientific research, and astronomy.^1^^,^^5^ Among these applications, medical imaging and diagnosis remain the most important applications of X-ray detection technologies. Clinical modalities such as radiography, mammography, fluoroscopy, and CT require detectors with high sensitivity, low noise, fast response, and low-dose operation to minimize radiation exposure while maintaining image quality.^1^^,^^5^^,^^8^^,^^10^^,^^12^ Flexible and wearable X-ray detectors are also attracting increasing interest for personalized healthcare and conformal imaging applications. Beyond healthcare, X-ray detectors are extensively used in industrial and security applications, including non-destructive inspection, structural defect analysis, baggage screening, and cargo inspection.^1^^,^^8^^,^^25^ In materials science research, X-ray diffraction, spectroscopy, and microscopy are indispensable for probing crystal structures, interfaces, and nanoscale phenomena.^1^^,^^8^^,^^25^ X-ray detectors also play key roles in astronomy, where they are used in space telescopes to study high-energy astrophysical events such as black holes, neutron stars, and supernova remnants.^1^ Table 1 summarizes these applications and their performance requirements.

**Table 1:** The main applications of X-ray detectors and their performance requirements

Applications		Energy range	Performance reequipments			Typical materials	Halide perovskites?
			Sensitivity (μC Gy_air_^−1^ cm^−2^)	Detection limit (nGy_air_ s^−1^)	Spatial resolution (lp mm^−1^)		
Medical imaging and radiotherapy	Digital radiography	40–120 keV	>10^2^–10^4^	<100–1,000	∼3–5	α-Se, CsI:Tl, GOS, a-Si	Yes
	Mammography	20–40 keV	>10^3^–10^5^	<50	>10	α-Se, CsI:Tl, a-Si	Yes
	CT	80–140 keV	>10^2^–10^3^	<1,000	∼5–10	CdWO_4_, GOS, CZT	Potentially
Security and Industry		50 keV–1 MeV	>10^2^–10^4^	<10^3^	High	CdZnTe (CZT), NaI:Tl, BGO	Yes
Materials research		1–100 keV	Moderate	Very low	Very high	Ge, Si, CZT	Potentially
Astronomy		0.1–100 keV	High	Ultra-low	Moderate	Si, CdTe, CZT	Attractive

Abbreviations: a-Si, amorphous silicon; GOS, Gd_2_O_2_S; BGO, Bi_4_Ge_3_O_12_; CZT, CdZnTe.^2^^,^^8^^,^^9^^,^^10^^,^^11^^,^^12^

#### Figures-of-merit for X-ray imaging systems

The performance of X-ray imaging systems is commonly evaluated using several important figures-of-merit, mainly including detective quantum efficiency (DQE), spatial resolution and modulation transfer function (MTF), energy resolution, dynamic range, contrast resolution, imaging noise, distortions, and artifacts.^1^^,^^5^^,^^29^^,^^31^^,^^32^ DQE describes how effectively an imaging system converts incident X-ray photons into useful image information while preserving the SNR, being defined as: DQE(*f*) = [SNR_out_(*f*)/SNR_in_(*f*)]^2^, where *f* is the spatial frequency (Figure 1F).^5^^,^^32^ A higher DQE enables improved image quality at a given radiation dose or, alternatively, reduced patient exposure without sacrificing imaging performance.^32^ Spatial resolution characterizes the ability of an imaging system to resolve fine structural details and is commonly quantified by the MTF, which describes the preservation of image contrast as a function of spatial frequency (*f*) (unit: line pairs per millimeter = lp mm^−1^) (Figure 1G).^31^^,^^32^^,^^33^ High spatial resolution is particularly important for applications requiring accurate identification of small features, such as mammography and microstructural inspection. Dynamic range defines the range of detectable X-ray intensities over which an imaging system can maintain accurate signal output.^32^^,^^33^ A wide dynamic range allows simultaneous visualization of regions with both strong and weak absorption, improving imaging versatility and diagnostic reliability.^32^ Contrast resolution represents the ability to distinguish small differences in attenuation between adjacent regions and is strongly influenced by detector sensitivity, noise level, and image-processing capability.^31^^,^^33^ Its value can be calculated as: CR = (*I*_b_ – *I*_0_)/(*I*_b_ + *I*_0_), where *I*_b_ and *I*_0_ represent the different intensities of the background and the target object. Imaging noise defines the unwanted variations of signal not related to the object being imaged.^31^^,^^32^^,^^33^ Low imaging noise is essential for achieving high-quality images, especially under low-dose conditions. Noise can originate from both quantum fluctuations in X-ray photons and electronic noise from detector components and readout circuits. In addition, Image distortions and artifacts caused by defective pixels, scattering effects, or mechanical deformation can negatively affect imaging accuracy and diagnostic interpretation.^31^^,^^33^

Overall, advanced X-ray imaging systems require high sensitivity, efficient charge collection, fast response, low noise, and excellent spatial and contrast resolution. These stringent requirements are driving the exploration of next-generation semiconductor materials and device architectures. In this context, halide perovskites and related heterostructure engineering strategies have emerged as highly promising approaches for developing low-dose, high-resolution, and flexible X-ray imaging technologies.

## Application-driving features of halide perovskites for X-ray detectors

Halide perovskites have emerged as highly promising materials for X-ray detection owing to their unique combination of excellent physical characteristics, structural versatility, and low-cost processing. Compared with conventional semiconductor materials, halide perovskites offer strong X-ray absorption, high *μτ* products, tunable electronic structures, low trap densities, high resistivity, and excellent scintillation properties, making them attractive for both direct and indirect X-ray detectors.^8^^,^^10^^,^^17^

### Adjustable structure with tuneable bandgap

**Figure 2:**
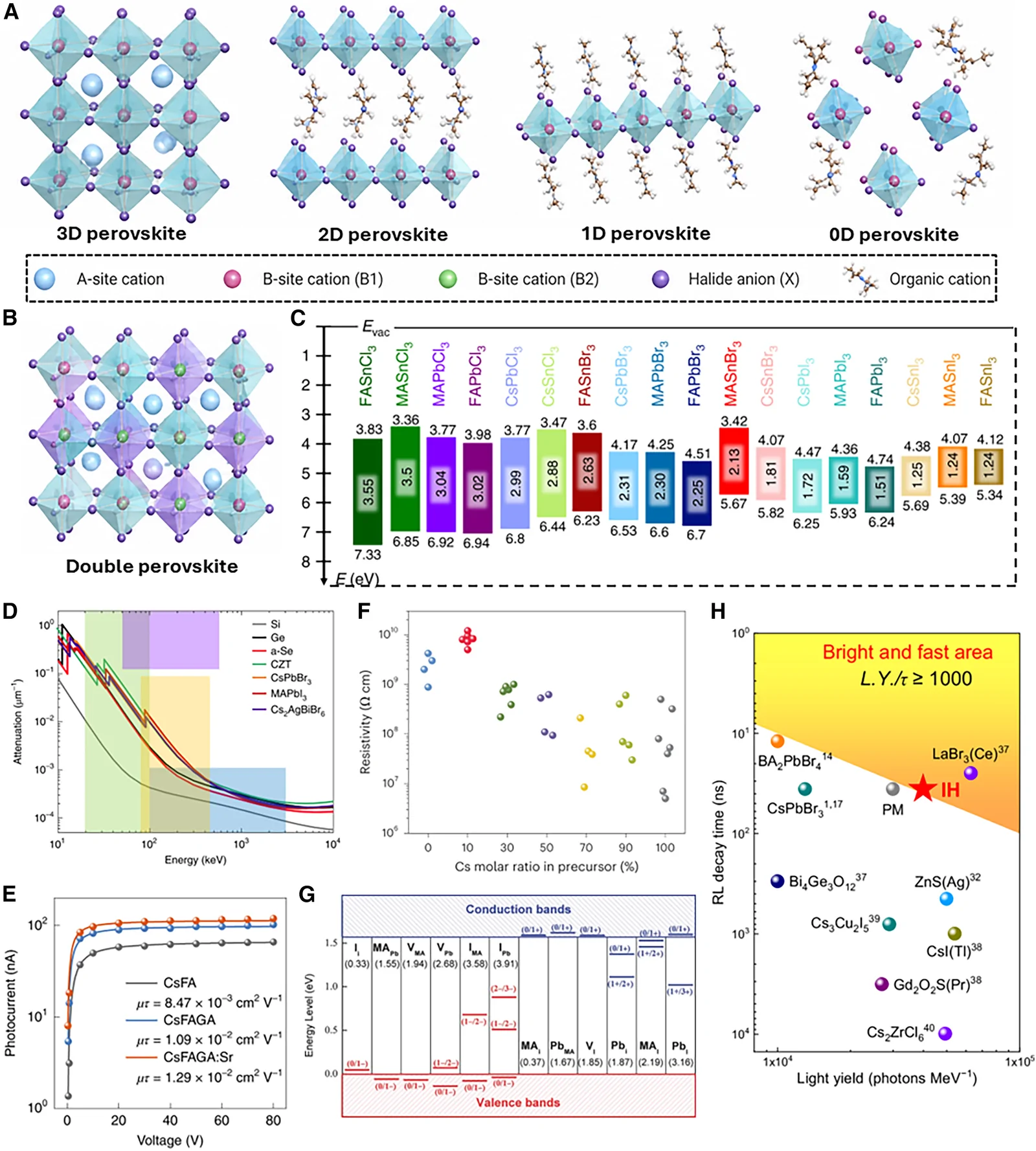
Halide perovskites with different structures and promising features (A) Schematically showing the crystal structures of typical 3D, 2D, 1D, and 0D halide perovskites. (B) Schematical illustration of double perovskite. (C) Schematic energy level diagram of various typical halide perovskites with tunable bandgaps as well as different VB and CB values.^34^ (D) Linear attenuation coefficient of CsPbBr_3_, MAPbI_3_, Cs_2_AgBiBr_6_, Si, Ge, α-Se, and CdTe versus photon energy.^12^ (E) Photoconductivity Pb-based single crystals with different A-site dopings, along with their μτ products.^36^ (F) The resistivity of FA_x_Cs_1–x_PbBr_3_ single crystals grown from solution with different cesium molar ratios.^37^ (G) The transition energy levels of all the possible intrinsic defects (acceptors/donors) in MAPbI_3_ hybrid perovskite.^17^ (H) Comparison of perovskite and commercial scintillators in terms of RL decay time and light yield. LY/τ exceeding 1,000 is defined as the bright and fast area. The numbers beside the names of scintillators are the cited references therein.^38^ Reproduced with permission (C–H).

Halide perovskites generally adopt the ABX_3_ crystal structure, where A is an organic group or inorganic cation (e.g., MA^+^, FA^+^, or Cs^+^), B is a divalent metal cation (typically Pb^2+^ or Sn^2+^), and X is a halide anion (I^−^, Br^−^, Cl^−^, or their mixtures).^12^^,^^34^^,^^35^ Their structures and dimensionalities can be readily tailored through compositional engineering, enabling the formation of 3D, 2D, and even one-dimensional (1D) and zero-dimensional (0D) perovskites (Figure 2A).^12^ In addition, Pb-free double perovskites (such as Cs_2_AgBiBr_6_ or Cs_2_TeI_6_) and Bi-based perovskite derivatives (like 0D Cs_3_Bi_2_I_9_) have attracted increasing attention because of their improved environmental compatibility and structural stability (Figure 2B).^12^ More importantly, the compositional flexibility of halide perovskites enables tunable bandgaps spanning around 1.2–3.2 eV, allowing optimization of carrier transport, resistivity, and radiation response for different X-ray detection applications (Figure 2C).^34^

### Large attenuation coefficient

One of the most important advantages of halide perovskites is their strong X-ray attenuation capability. The presence of heavy elements such as Pb, Sn, I, or Bi provides high average atomic numbers and large attenuation coefficients, enabling efficient absorption of high-energy photons.^1^^,^^5^^,^^25^ For example, CsPbBr_3_ possesses an average atomic number comparable with or even higher than that of commercial CdZnTe, while MAPbI_3_ and CsPbI_3_ exhibit attenuation coefficients competitive with widely used detector materials such as Si, Ge, α-Se, and CdTe (Figure 2D).^17^^,^^25^ These properties allow efficient X-ray absorption even in relatively thin active layers.

### High *μτ* product

Halide perovskites also exhibit excellent charge transport properties. Their low trap densities and defect-tolerant electronic structures contribute to large *μτ* products, which facilitate efficient carrier extraction and suppress recombination losses.^5^^,^^17^^,^^25^ For example, single-crystal MAPbBr_3_ and MAPbI_3_ have demonstrated the high *μτ* products on the order of 1.4 × 10^−2^ cm^2^ V^−1^, together with ultralong carrier diffusion lengths and high internal quantum efficiencies (Figure 2E).^17^^,^^36^^,^^39^^,^^40^ Such characteristics are highly beneficial for achieving high sensitivity and efficient charge collection in direct X-ray detectors.

### Large bulk resistance and radiation hardness

Large bulk resistivity is another key advantage, as it suppresses dark current and reduces electronic noise. Depending on composition and dimensionality, halide perovskites can exhibit resistivities ranging from 10^7^ to 10^12^ Ω cm (Figure 2F).^37^^,^^41^^,^^42^^,^^43^ In particular, low-dimensional and Pb-free perovskites (like Cs_2_AgBiBr_6_ and Cs_3_Bi_2_I_9_ single crystals) often show enhanced resistivity and improved environmental stability, making them attractive for low-dose X-ray detection.^10^ Another notable feature is their excellent radiation tolerance, which refers to the stability or repairability after being exposed to high dose or continuous X-ray radiation. Unlike many conventional semiconductors, halide perovskites possess remarkable defect tolerance and, in some cases, self-healing behavior under prolonged irradiation (Figure 2G).^17^^,^^44^ These characteristics contribute to stable detector performance under continuous X-ray exposure.

### High LY

Halide perovskites can serve as promising scintillator materials for indirect X-ray imaging because of their high LYs, fast radioluminescence decay, and tunable emission wavelengths (Figure 2H).^38^ Perovskite nanocrystals and quantum dots can achieve efficient and spectrally adjustable luminescence, which is advantageous for coupling with commercial photodetectors.^45^^,^^46^

### Low-cost raw materials and facile processing

Halide perovskites can be synthesized using low-temperature solution-processing methods under conditions closer to room temperature (<150 °C), like inverse temperature crystallization (ITC), antisolvent vapor-assisted crystallization (AVC), hot injection method, and supersaturated recrystallization,^10^^,^^14^^,^^15^ which are also beneficial for commercial X-ray detecting and imaging devices. Various raw materials, which are utilized for synthesizing halide perovskites, are rich in nature and are low cost; it is essential for their commercialization. According to main suppliers including Aladdin and Sigma-Aldrich, the entire material cost for halide perovskites is estimated to be in the range of USD2.5–USD3.7 cm^−3^, which is much lower than those of traditional materials for the commercial scintillators, such as YAG:Ce (USD25 g^−1^), LYSO (USD8.4 g^−1^), and YAP:Ce (USD60 g^−1^).^17^^,^^25^ The low cost for synthesizing high-quality halide perovskite samples is also comparable with that for preparing CsI:Tl (USD2.5 g^−1^).^17^^,^^25^ Once the production for halide perovskites scales up for practical applications, their material cost of high-performance X-ray detectors may decrease by 5–10 times. Thus, the total projected cost for the growth of perovskite single crystals is calculated as < USD0.3 cm^−3^, which is much (3/4 times) cheaper than the commercial semiconducting materials, like CdZnTe (CZT) crystals (USD0.9–USD1.2 cm^−3^).^17^^,^^25^

In addition, compared with the widely used commercial semiconductors such as Si, Se, and CZT, of which the growth processes typically require high temperature, high vacuum, and/or complex instruments, halide perovskites are usually produced by solution-processed methods under the conditions closer to room temperature (<150 °C).^1^^,^^10^ In addition, the related detection devices can be easily fabricated through one-step spin-coating or spray method and directly using the freshly synthesized halide perovskite single crystals, which offers a large operation room for further integration.

## Perovskite-based heterostructures

### Fundamentals of heterostructure engineering

Semiconductor heterostructures are fundamental building blocks in modern opto-/electronic and energy devices, where interfaces govern charge separation, transport, recombination, and energy transfer. A heterostructure is formed when two materials with different electronic structures are brought into contact, generating an interface region that can introduce built-in electric fields and energy barriers. In halide perovskite X-ray detectors, heterostructure engineering has emerged as an effective strategy for improving sensitivity, suppressing dark current, enhancing charge-collection efficiency, and stabilizing device operation.^47^^,^^48^

**Figure 3:**
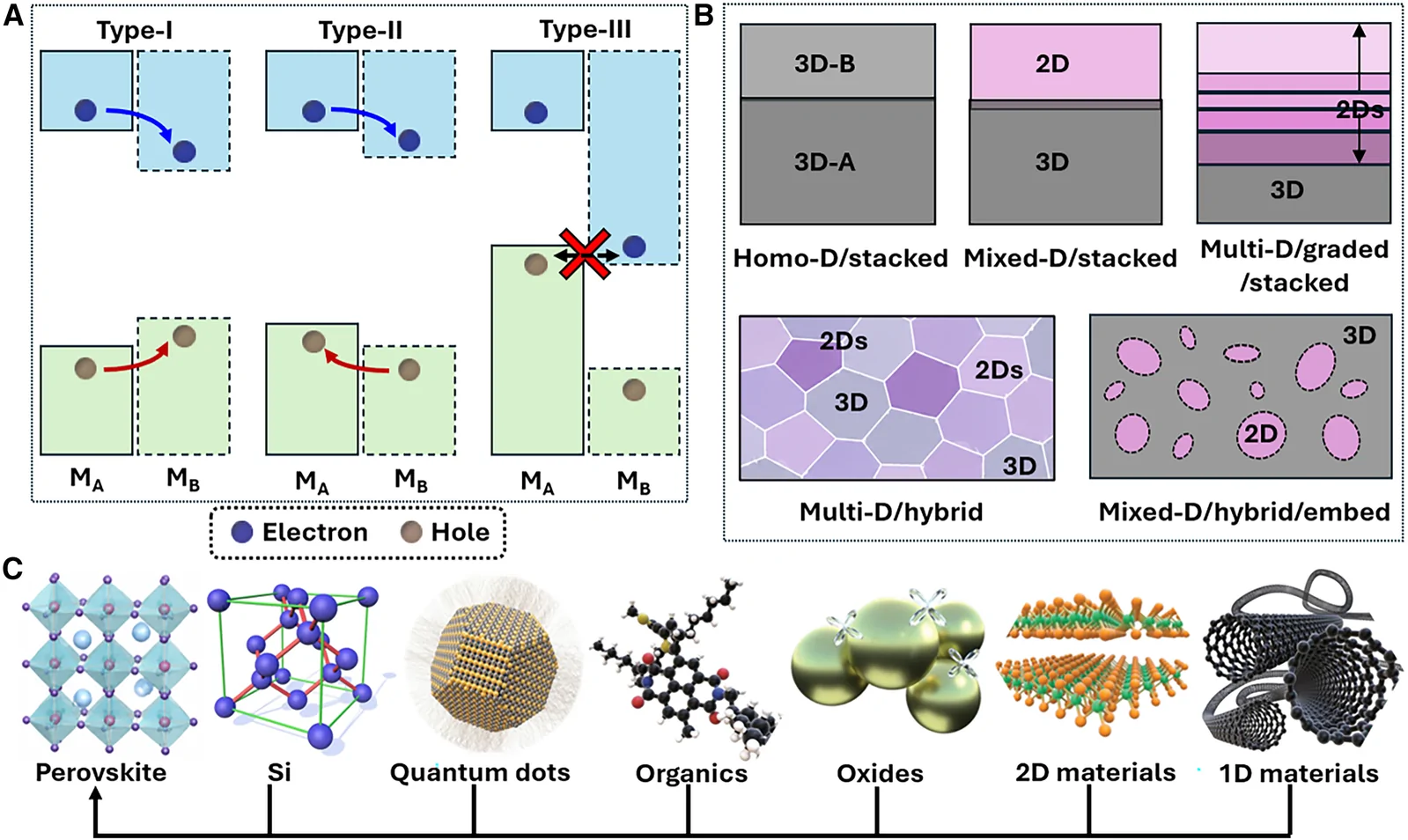
Perovskite-based heterostructures (A) Three types of heterojunctions (M_A_ and M_B_ are materials A and B, respectively) along with their bang alignments and charge transfer processes. (B) Various types of perovskite-perovskite heterostructures, including homo-/mixed-dimensional (D) stacked, multi-D/graded/stacked, multi-D/hybrid, and mixed-D/embed heterostructures. (C) Perovskite-based heterostructures with other functional materials such as Si, metal oxides, organic semiconductors, 2D systems such as graphene and transition metal dichalcogenides, 1D materials like CNTs, and 0D materials like quantum dots.^47^ Reproduced with permission (D).

Based on energy-band alignment, semiconductor heterojunctions are generally classified into three types (Figure 3A). In type I heterojunctions, the conduction band minimum (CBM) and valence band maximum (VBM) of one semiconductor are both located within the bandgap of the other. Such band alignment confines both electrons and holes in the same material and is therefore favorable for radiative recombination and light-emission applications. In type II heterojunctions, the CBM and VBM are staggered, enabling spatial separation of electrons and holes across the interface. This charge-separation effect is highly beneficial for photodetectors and X-ray detectors because it suppresses carrier recombination and promotes efficient charge extraction. Type III heterojunctions exhibit broken-gap alignment and are mainly relevant for tunneling-based devices.

Beyond band alignment, heterostructure performance is strongly influenced by interface chemistry, defect states, and electrostatic potential distribution. In halide perovskites, the coexistence of ionic and electronic transport introduces additional complexity, where ion migration, defect accumulation, and interfacial polarization can dynamically reshape band profiles.^48^ In detail, under an applied electric field or light illumination, mobile ionic species (for example, halide ions, vacancies, or organic cations) can accumulate near the related interfaces, leading to the formation of space-charge regions and internal electric fields. These ionic redistributions alter the local band bending, energy-level alignment, and charge injection/extraction barriers at the relevant heterointerface. In addition, interfacial polarization arising from charge accumulation or dipole formation can further modulate the interfacial potential landscape, resulting in a dynamic evolution of the band structure during device operation. Therefore, rational heterostructure design must consider not only static band alignment but also interfacial defect passivation, ion-blocking capability, and long-term stability under bias and irradiation.

### Formation of perovskite-based heterostructures

Halide perovskites provide unique opportunities for heterostructure engineering owing to their compositional tunability, defect tolerance, and mixed ionic-electronic transport properties. Both perovskite-perovskite and perovskite-other material heterostructures have been widely explored to tailor interfacial energetics, carrier dynamics, and ionic transport, thereby simultaneously improving detector sensitivity, operational stability, and imaging performance.

Perovskite-perovskite heterostructures commonly include vertical architectures, such as 3D/3D homo-dimensional junctions, 3D/2D mixed-dimensional stacked structures, multi-dimensional graded-composition junctions, multi-dimensional hybrids, and embed hybrids (Figure 3B).^49^ In these systems, the wide-bandgap low-dimensional perovskites can serve as surface passivation or barrier layers, while the 3D perovskites provide efficient carrier transport pathways. Such vertical heterostructures simultaneously enhance environmental stability, suppress ion migration, and maintain high charge mobility. Compositionally graded perovskite junctions further introduce internal electric fields that facilitate carrier separation and reduce recombination losses across thick active layers. Among these architectures, 2D/3D perovskite heterostructures have attracted particular attention because the ultrathin 2D perovskite layer simultaneously enhances environmental stability and regulates interfacial carrier transport. The hydrophobic organic spacer cations in the 2D perovskite effectively impede moisture penetration while serving as a physical barrier that suppresses the migration of mobile ionic species, such as halide vacancies and organic cations, from the underlying 3D perovskite. Consequently, ion accumulation at interfaces and electrodes is significantly reduced, leading to suppressed current drift, lower hysteresis, and enhanced operational stability under continuous electrical bias and X-ray irradiation.^49^ In addition, the well-defined layered crystal structure of 2D perovskites effectively passivates under-coordinated Pb^2+^ ions, halide vacancies, and other surface defects at the 2D/3D interface, thereby reducing trap-assisted non-radiative recombination and improving carrier lifetime. Perovskite-other material heterostructures expand the design space by integrating perovskites with functional materials such as metal oxides (e.g., TiO_2_, SnO_2_), organic semiconductors, carbon-based materials, and low-dimensional systems including graphene and transition metal dichalcogenides (Figure 3C).^47^ These heterostructures can form selective charge-transport pathways, reduce interfacial recombination, and suppress leakage currents. For instance, wide-bandgap oxides or polymer interlayers can act as carrier-blocking layers to lower dark current, while 2D materials with high carrier mobility and atomically sharp interfaces enable efficient charge extraction and reduced noise. In addition, hybrid composites—such as perovskite/quantum dot or perovskite/polymer systems—can improve mechanical flexibility, enhance radioluminescence, and enable large-area processing.

Importantly, the heterointerfaces at the microscopic level in perovskite heterostructures are governed by diverse interactions, including ionic bonding, covalent coordination, hydrogen bonding, and van der Waals forces. Surface ligands and interfacial modifiers (e.g., Lewis bases/acids, ammonium salts, and small molecules) play a crucial role in passivating defects, improving crystallinity, suppressing nonradiative recombination, and enhancing electronic coupling. These interactions not only reduce trap-assisted recombination but also regulate ion migration pathways and interfacial polarization. Notably, heterostructure engineering in halide perovskite X-ray detectors extends beyond conventional band alignment. Carefully designed interfaces can introduce built-in electric fields, confine carriers, suppress ionic drift, and mitigate noise under prolonged bias and irradiation. In addition, forming heterostructures could effectively mitigate key degradation pathways, such as moisture ingress, thermal decomposition, ion migration under bias, and radiation-induced defect accumulation under X-ray irradiation, and thus improve device stability by blocking ion migration pathways, reducing trap formation, and enhancing interfacial chemical and structural robustness. As a result, understanding the interplay between interface chemistry, band alignment, and coupled ionic-electronic processes is essential for developing high-performance, stable, and application-ready perovskite X-ray detectors.

## Heterostructure engineering for advancing X-ray detectors

### Perovskite-based heterostructure as active X-ray absorbers

The performance of direct and indirect X-ray detectors is fundamentally determined by the X-ray absorption capability, carrier transport properties, and operational stability of the active materials. Although halide perovskites exhibit intrinsically favorable properties, including large attenuation coefficients, high carrier mobility-lifetime (*μτ*) products, defect tolerance, and solution processability, single-component perovskite layers still suffer from limitations such as leakage current, ion migration, trap-assisted recombination, and environmental instability. Heterostructure engineering has therefore emerged as a powerful strategy for simultaneously regulating band alignment, carrier dynamics, defect distribution, and interfacial energetics, thereby improving X-ray detection performance and operational stability.

In recent years, a wide range of perovskite-based heterostructures has been developed, including perovskite/perovskite junctions, mixed-dimensional heterostructures, and hybrid architectures integrating perovskites with other semiconductors or low-dimensional materials. Depending on the band alignment and interface design, these heterostructures can generate built-in electric fields that facilitate carrier separation and extraction, suppress charge accumulation, reduce dark current, and mitigate ion migration under electrical bias and continuous irradiation. In addition, heterostructures can improve crystal quality and passivate interfacial defects, which is particularly important for thick-film or single-crystal X-ray absorbers.

#### Perovskite-perovskite heterostructures

Among the various strategies, perovskite-perovskite heterostructures are especially attractive because they preserve the favorable optoelectronic properties and structural compatibility of halide perovskites while enabling tunable band structures and interface functionalities.^25^ By combining perovskites with different bandgaps, carrier types, or dimensionalities, heterojunctions with tailored charge-transport behavior can be realized.

**Figure 4:**
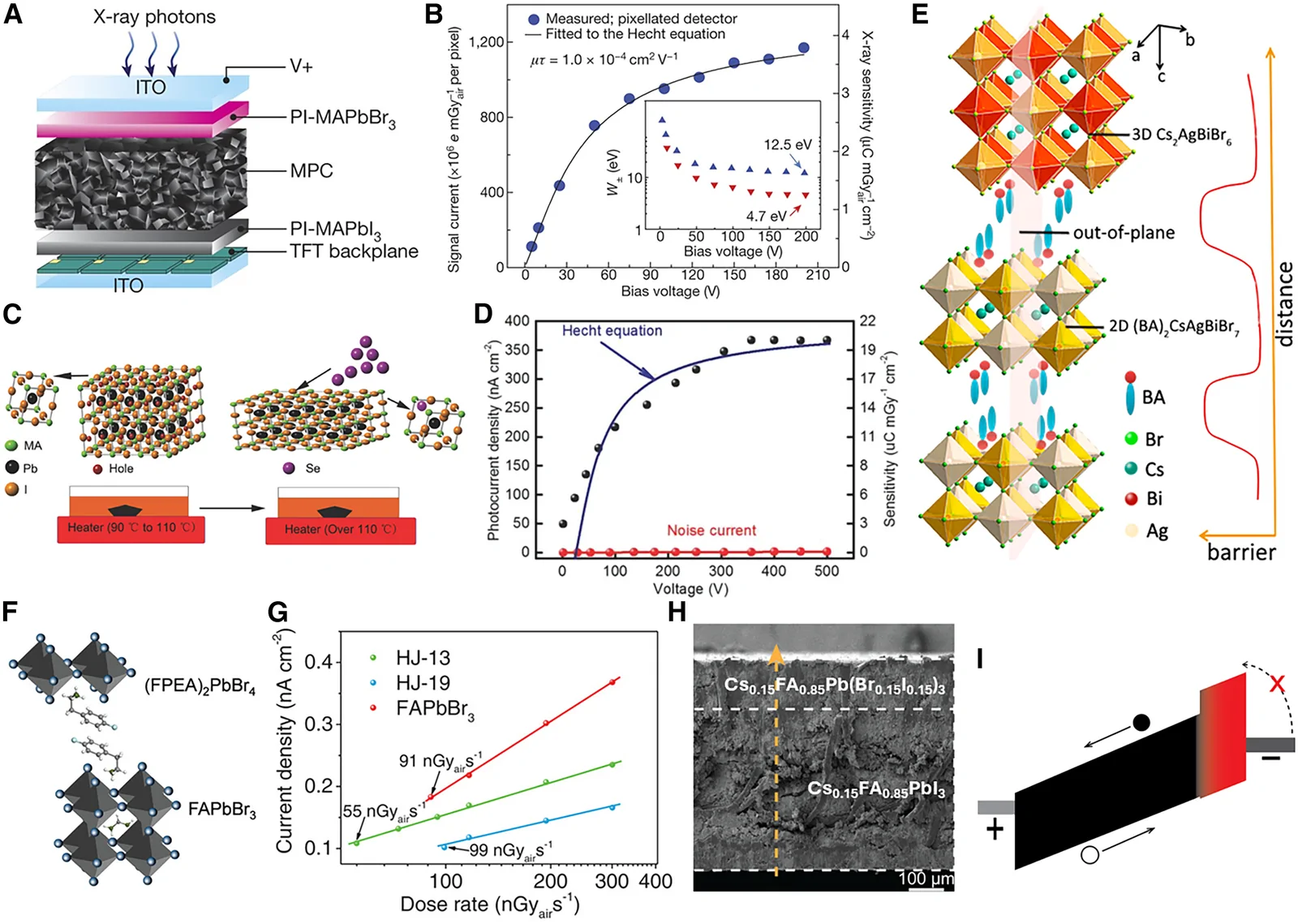
Perovskite-perovskite heterostructures as active mediums (A) Illustration of an all-solution-processed digital X-ray detector containing various perovskites layers.^50^ (B) Charge collection and sensitivity characteristics of the MPC detector measured at 100 kVp. The inset shows W± in the pixelated (blue symbols) and diode (red symbols) detectors.^50^ (C) Illustration of crystal structure when doped MAPbI_3_ single crystals are grown at temperatures under and above 110 °C, respectively.^51^ (D) Photocurrent and sensitivity as functions of voltage for the doped MAPbI3 PSC device radiated by 60 keV X-ray photons.^51^ (E) Structural illustration of the 2D/3D perovskite (BA)_2_CsAgBiBr_7_/Cs_2_AgBiBr_6_ heterocrystal.^52^ (F) The structural illustration of 3D/2D single-crystal perovskite heterostructure of FAPbBr_3_/(FPEA)_2_PbBr_4_.^53^ (G) The current density of 3D/2D perovskite single-crystal heterojunction and FAPbBr_3_ single-crystal devices at different X-ray dose rates, respectively.^53^ (H) Cross-sectional SEM image of the heterojunction perovskite film with the structure scheme of Cs_0.15_FA_0.85_PbI_3_/Cs_0.15_FA_0.85_Pb(I_0.15_Br_0.85_)_3_.^54^ (I) Band diagram of the formed heterojunction perovskite film.^54^ Reproduced with permission (A–I).

One important direction involves constructing p-n junctions or type II heterojunctions within perovskite absorbers to suppress leakage current and enhance carrier extraction, so as to reduce the detection limit of X-ray detectors and also reduce the X-ray dose during the imaging process. For example, Kim et al. introduced an interfacial engineering technique to modify the work function by tactfully employing polyimide (PI)-MAPbBr_3_ composites and PI-MAPbI_3_ composites as hole-blocking layer and hole-transporting layer, respectively (Figure 4A).^50^ The optimized X-ray detector based on MAPbI_3_ thick film possessed an impressive sensitivity reaching up to 11 μC mGy_air_^−1^ cm^−2^ (Figure 4B).^50^ Moreover, the solution-based and facile fabrication process makes the devices easily integrated into readout circuits, enabling low-dose, low-cost practical X-ray imaging applications. In addition, Wang et al. engineered a *p-n* homojunction in MAPbI_3_ single crystals through controlled selenium (Se) doping. The pristine MAPbI_3_ acted as a *p*-type semiconductor, whereas Se incorporation partially substituted I^−^ ions and introduced *n*-type behavior (Figure 4C).^51^ Thanks to the materials design, the optimized junction generated an internal electric field that promoted charge separation and reduced carrier recombination. The resulting X-ray detectors exhibited a low dark current density of 4 nA cm^−2^ at an electric field of −100 V cm^−1^ together with a sensitivity of 21 μC Gy_air_^−1^ cm^−2^ (Figure 4D).^51^ Such composition-engineered perovskite homojunctions provide an effective route toward low-noise X-ray detectors.

Mixed-dimensional perovskite heterostructures have also attracted substantial attention. Notably, 3D perovskites generally possess superior carrier mobility and show high sensitivity to X-rays, whereas low-dimensional perovskites like 2D perovskites could exhibit enhanced moisture resistance, suppressed ion migration, and larger resistivity. Combining these advantages within a single mixed-dimensional heterostructure could enable the optimal device performance, thereby reducing the detection limit dose rate and maintaining the high sensitivity to X-rays. Zhang et al. reported high-quality 2D/3D Pb-free perovskite hetero-crystals based on (BA)_2_CsAgBiBr_7_/Cs_2_AgBiBr_6_ synthesized through the solution-processed *in situ* heteroepitaxial approach (Figure 4E).^52^ The resulting heterogeneous single-crystal X-ray detectors working in a self-powered mode could achieve a promising sensitivity of 206 μC Gy^−1^ cm^−2^, whereas the pristine Cs_2_AgBiBr_6_ single-crystal detector just serves as a baseline device with relatively low sensitivity (approximately 4.2 μC Gy^−1^ cm^−2^) due to the limited charge-collection efficiency.^52^ Furthermore, the devices exhibited excellent operational stability, maintaining stable photocurrent and dark current outputs over 40 consecutive X-ray on/off switching cycles. More importantly, the detectors showed a highly stable baseline, with the dark current remaining essentially unchanged after 3 h of continuous operation. The photoresponse also demonstrated excellent ambient stability, with no noticeable degradation after storage in air for 20 days^52^ Similarly, He et al. introduced long-chain organic material—4-fluorophenethylammonium (FPEA) cations to replace organic-group FA^+^ ions and constructed a large-sized 3D/2D perovskite heterojunction through facile solution-processed epitaxial growth method (Figure 4F).^53^ As for the reference X-ray detector, the pristine 3D CsPbBr_3_ single-crystal device exhibits intrinsically good X-ray response due to its excellent charge transport properties; however, it suffers from relatively high dark current and noticeable baseline drift under continuous operation, which are attributed to pronounced ion migration and surface-related defect states.^53^ The inserted 2D perovskite layer effectively suppressed ion migration and interfacial recombination while maintaining efficient carrier transport in the 3D bulk region. The X-ray detectors based on such 3D/2D perovskite heterojunctions exhibited the lowest detectable dose rate of 55 nGy_air_ s^−1^ to hard X-rays (Figure 4G).^53^ Notably, the heterojunction devices also improved long-term stability. Under continuous X-rays irradiation under a huge X-ray dose rate of 130 μGy_air_ s^−1^, the device could absorb X-rays with a total dose of 0.66 Gy_air_ and still show good operation stability.^53^

Besides epitaxial structures, perovskite heterojunctions can also be realized through compositional grading or laminated architectures. Recently, Zhou et al. fabricated flexible heterojunction X-ray detectors by integrating Cs_0.15_FA_0.85_PbI_3_ and Cs_0.15_FA_0.85_Pb(I_0.15_Br_0.85_)_3_ layers within porous nylon matrices (Figure 4H).^54^ Owing to the staggered band alignment between the two perovskites (Figure 4I), the heterostructure effectively suppressed leakage current while maintaining efficient charge transport.^54^ The pristine polycrystalline perovskite X-ray detectors exhibited high sensitivity (approximately 2.0 × 10^3^ μC Gy_air_^−1^ cm^−2^) but suffered from a relatively large dark current (approximately 10^–7^ A cm^−2^), which limits its SNR. In contrast, the optimized heterojunction devices significantly suppress dark current by more than two orders of magnitude (approximately 10^–9^ A cm^−2^) while exhibiting a remarkably low detection limit of 13.8 ± 0.29 nGy_air_ s^−1^ under 40-keV X-ray irradiation.^54^ Significantly, the heterojunction detectors exhibited excellent operational stability, showing negligible dark-current drift during 7 h of continuous biasing and maintaining stable performance after 1518 min of continuous 60-keV X-ray irradiation, corresponding to an accumulated dose of 345.8 Gy_air_. Accelerated aging experiments and diffusion simulations further indicated that the heterojunction architecture is tolerant to halide interdiffusion, with a predicted operational lifetime exceeding 15 years.^54^ These results demonstrate that rationally designed perovskite heterostructures can substantially improve sensitivity and SNR while enabling mechanically flexible detectors and highly improving the operational stability.

#### Heterostructures between perovskite and other functional materials

Beyond perovskite-perovskite interfaces, integrating halide perovskites with other functional materials provides additional opportunities for regulating charge transport, defect passivation, and device integration. Various semiconductors, including organic semiconductors, metal oxides, and low-dimensional materials, have been incorporated into perovskite heterostructures for high-performance detection devices working in UV-visible-near-infrared range.^47^ As for X-ray detection applications, perovskite-based heterostructures with silicon and various quantum dots and nanocrystals have been demonstrated to be promising active layers for the enhanced performance.

**Figure 5:**
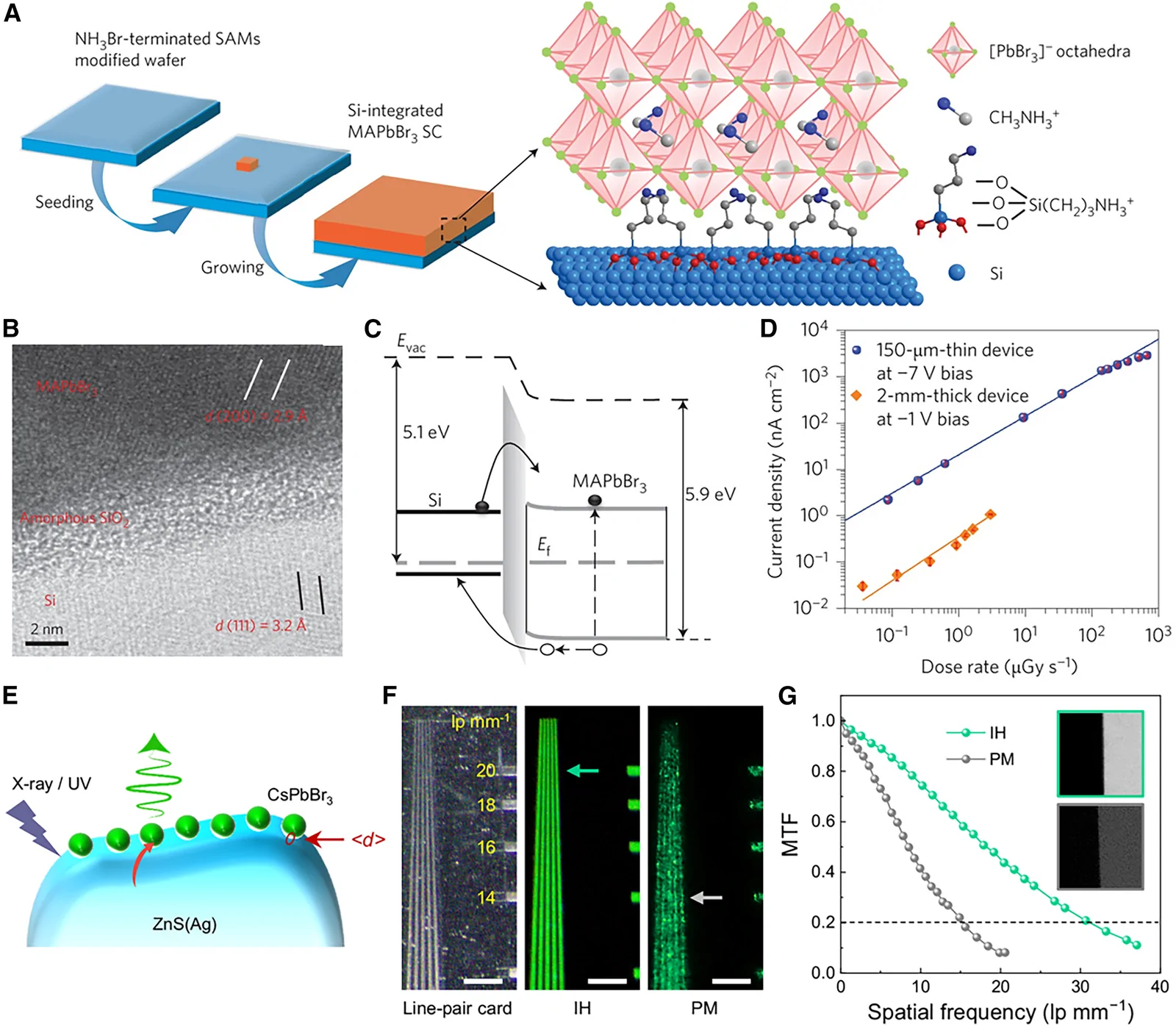
Perovskite-based heterostructures with other functional materials (A) Schematic illustration of the fabrication of Si-integrated MAPbBr3 single crystals (SCs) (not to scale—the silane thickness is highly exaggerated for clarity).^55^ (B) High-resolution TEM image of the cross-section interface of the Si-integrated MAPbBr_3_ single crystal.^55^ (C) Energy level diagram for the interface of the Si/MAPbBr_3_ single crystal with the dipole layer.^55^ (D) X-ray-generated photocurrent versus dose rate.^55^ (E) Structural configuration of the *in situ* synthesized heterostructure (IH-type) CsPbBr_3_-ZnS(Ag) nanocrystal serving as scintillators. <*d*> is the short average distances between CsPbBr_3_ nanocrystals and ZnS(Ag) microcrystal.^38^ (F) X-ray imaging spatial resolution of the IH-type and PM (physically mixed)-type CsPbBr_3_ scintillators determined by a standard line-pair card. Scale bars, 0.5 mm.^38^ (G) Modulation transfer function (MTF) curves, the insets are corresponding X-ray edge images.^38^ Reproduced with permission (A–G).

Silicon/perovskite heterostructures are particularly attractive because they combine the mature processing technologies of Si electronics with the superior X-ray absorption capability of halide perovskites. Wei et al. used a brominated (3-amino-propyl) triethoxysilane (NH_3_Br) molecular thin layer to mechanically and electrically integrate MAPbBr_3_ bulk single crystals onto Si wafer substrates, where the Si wafer could both serve as electrode and form high-quality heterojunction with MAPbBr_3_ single crystals (Figure 5A).^55^ The interfacial molecular layer improved both adhesion and electronic coupling between the perovskite and Si substrate, enabling efficient charge extraction and ultrahigh X-ray sensitivity under low-dose irradiation (Figure 5B).^55^ Compared with the pristine single-crystal devices, which are limited by relatively higher leakage current and interface-induced noise, the insertion of NH_3_Br molecular layer and the heterogeneous integration between single-crystal perovskite and Si wafer resulted in a significant reduction in dark current at higher bias (Figure 5C), which is critical for the Si-integrated MAPbBr_3_ single crystal detectors to sense a very low X-ray (8 keV) dose rate of <0.1 μGy_air_ s^−1^ with a high sensitivity of 2.1 × 10^4^ μC Gy_air_^−1^ cm^−2^ (Figure 5D).^55^ More importantly, such simple method for connecting perovskite single crystals onto commonly used substrates enable the easy, cost-effective, and scalable route for integrating perovskite X-ray detectors with established CMOS readout circuits, helpful for the practical applications in medical imaging and security checks.

Halide perovskite quantum-dot and nanocrystal heterostructures have also been explored. Yang et al. designed and *in situ* synthesized heterostructure (IH-type) multi-site ZnS(Ag)-CsPbBr_3_ to modulate the bright and fast features of scintillators (Figure 5E).^38^ The authors observed that the external energy from ZnS(Ag) could effectively transfer to CsPbBr_3_ based on the non-radiative Förster resonance energy transfer, resulting in a LY of 40,000 photons MeV^−1^. By combining a radioluminescence decay time of 36 ns and a spatial resolution of 30  lp mm^−1^ (Figures 5F and 5G), the scintillator enables high-speed X-ray imaging at 200 frames per second.^38^ Additionally, the heterostructured scintillator also exhibited excellent stability. The radioluminescence intensity remained nearly unchanged after storage in ambient air for 6 months and showed negligible degradation after exposure to a cumulative X-ray dose of 69 Gy_air_, demonstrating good air-storage and radiation stability of the scintillator.^38^ This study showcases the structure design is significant for obtaining bright and fast perovskite scintillators for the real-time X-ray imaging.

Overall, heterostructure engineering has become one of the most effective approaches for advancing perovskite X-ray absorbers. Through synergistic regulation of band alignment, defect distribution, interfacial electric fields, and carrier dynamics, perovskite-based heterostructures can simultaneously enhance sensitivity, suppress leakage current, improve operational stability, and facilitate scalable device integration for next-generation X-ray imaging technologies.

### Device design: Interfacial engineering

Beyond perovskite active-layer optimization, device architecture and interface engineering play equally important roles in determining the performance of perovskite X-ray detectors. Since X-ray-induced charge carriers must be efficiently extracted while leakage current and noise are minimized, rational device design is essential for achieving high sensitivity, low detection limits, and stable long-term operation.

**Figure 6:**
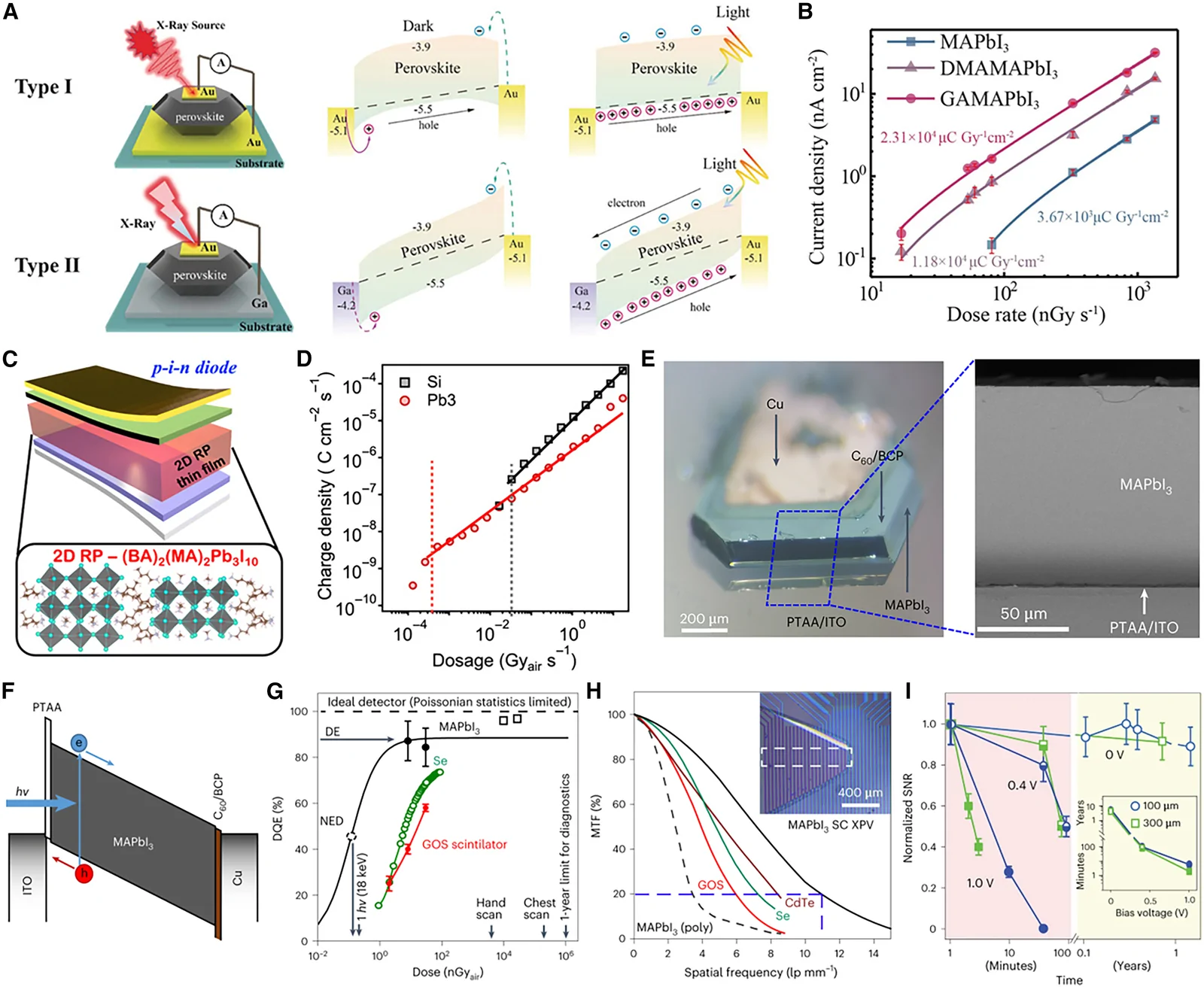
Heterointerfaces in X-ray detector devices (A) Architecture of the MAPbI_3_-based X-ray detector with using asymmetric electrodes (Au and Ga).^56^ (B) The GAMAPbI_3_ single-crystal device displaying the highest detector sensitivity of 2.3 × 10^4^ μC Gy_air_^−1^ cm^−2^ among the alloyed and unalloyed single crystals.^56^ (C) Schematic illustration of the 2D RP-based *p-i-n* thin-film X-ray detector device architecture composed of (BA)_2_(MA)_2_Pb_3_I_10_ (dubbed as Pb_3_) as an absorbing layer.^57^ (D) X-ray-induced charge density subtracted by the dark noise (signal-to-noise ratio) for 2D RP and silicon reference detector.^57^ (E) Photovoltaic-model X-ray detection device containing a 110-μm MAPbI_3_ single crystal and interfacial layers, along with its cross-sectional scanning electron microscopy image.^58^ (F) Energy band alignment and operation principle in the X-ray photovoltaic mode.^58^ (G) DQE dependencies on the dose for the MAPbI_3_ single-crystal X-ray photovoltaic detector and the gadolinium oxysulfide (GOS) scintillator.^58^ (H) MTF dependencies versus spatial frequency for MAPbI_3_ single-crystal X-ray photovoltaic device, polycrystalline MAPbI_3_ and commercial GOS, amorphous Se, and CdTe single-crystal detectors.^58^ (I) X-ray photovoltaic detection devices demonstrating stable single-photon-counting performance for over one year.^58^ Reproduced with permission (A–I).

In general, there are mainly two types of architectures for X-ray detectors (in particular the direct-model devices), including photoconductor-type and photodiode-type configurations. Photoconductors generally employ symmetric electrodes and operate under external bias, whereas photodiodes utilize asymmetric contacts to form built-in electric fields. Compared with symmetric architectures, asymmetric-contact devices can generate large Schottky barriers under reverse bias, thereby suppressing carrier injection and reducing dark current. This strategy has proven highly effective for improving SNR and detection sensitivity. For instance, asymmetric Ga/perovskite/Au-architecture direct-model X-ray detectors based on GAMAPbI_3_ perovskite single crystals (Figure 6A) exhibited a substantially lower dark current compared with symmetric-contact devices, resulting in the higher sensitivities up to 2.3 × 10^4^ μC Gy_air_^−1^ cm^−2^ under low operating voltage of −5 V (Figure 6B).^56^ Based on this idea, more research works have appeared to improve performance of direct X-ray detection devices. Similar approaches using metal electrodes with tailored work functions, including Al, Ag, Cr, Cu, and Ti/Au, have also been widely adopted to regulate Schottky barrier heights and optimize carrier extraction, aiming to gain the suppressed leakage current and optimized sensitivity.^52^^,^^54^

Interfacial charge-transport layers represent another key strategy for enhancing device performance. Similar to perovskite photovoltaics and photodetectors, electron-transport layers (ETLs) and hole-transport layers (HTLs) can selectively collect carriers, suppress recombination, and reduce leakage current. Organic ETLs such as PCBM, C_60_, BCP, or double layer of PCBM/C_60_ or BCP/C_60_), as well as inorganic ETLs like metal oxide layers (including ZnO or TiO_2_), have been extensively employed in perovskite X-ray detectors.^8^^,^^10^^,^^12^^,^^17^^,^^25^ Meanwhile, HTLs such as PTAA, PEDOT:PSS, or SpiroTTB have been employed in various perovskite X-ray detectors, with the aim of improving device performance and even device stability.^8^^,^^10^^,^^12^^,^^17^^,^^25^ For example, Nie et al. designed a new type of *p-i-n* junction X-ray detector with 2D RP-phase (BA)_2_(MA)_2_Pb_3_I_10_ film working as active layer (Figure 6C).^57^ In detail, poly[*bis*(4-phenyl)(2,4,6-trimethylphenyl)amine] (PTAA) layer as *p*-type contact and C_60_ layer as *n*-type contact were introduced to construct high-quality *p-i-n* junction configuration, helping for minimizing the dark current and noise, thus determining a high device detectivity (up to 0.276 C Gy_air_^−1^ cm^−3^) (Figure 6D) and a low detection limit as well as the promising device stability.^57^

Notably, carefully designed multilayer interfaces can enable photovoltaic-mode X-ray detection without the need for high external bias. Sakhatskyi et al. successfully fabricated MAPbI_3_ single-crystal X-ray detectors operated in the photovoltaic mode (Figure 6E); in detail, the work-function asymmetry of the used electrical contacts (through using PTAA as hole-transporting layer and C_60_/BCP bilayer as electron-transporting layer) plays a key role for creating strong work-function asymmetry (Figure 6F).^58^ The resulting X-ray detection devices operated in photovoltaic mode and demonstrated stable single-photon-counting performance; detection efficiency of 88% and noise-equivalent dose of 90 pGy_air_ are obtained with 18 keV X-rays, allowing single-photon-sensitive, low-dose and energy-resolved X-ray imaging (Figure 6G).^58^ Array detectors demonstrate high spatial resolution up to 11 lp mm−1 (Figure 6H).^58^ Such self-powered or low-bias operation is highly attractive for reducing power consumption and improving detector stability.

Interfacial engineering can also regulate ion migration and interfacial polarization, which are critical issues in halide perovskites under continuous electrical bias and irradiation. The accumulation of mobile ions near interfaces can induce baseline drift, increase noise current, and deteriorate operational stability. For instance, owing to the self-powered or zero-bias operation based on the particular interfacial design, the optimal devices retain their characteristics for at least one and a half years (in air, without encapsulation), with the projected device half-life (time that corresponds to a 50% drop in the normalized SNR) of several years (Figure 6I).^58^ Introducing suitable interlayers or passivation layers can also suppress ionic migration pathways and stabilize interfacial electric fields. For example, polymer interlayers, self-assembled molecules, and low-dimensional perovskite surface layers have all been shown to reduce defect-assisted ion migration and improve device reproducibility and long-term stability.

In addition, heterostructure interfaces can influence crystal growth and film morphology. Interfacial modifiers often promote larger grain sizes, improved crystallinity, and reduced pinhole density, which collectively contribute to enhanced carrier transport and lower trap-assisted recombination.^59^^,^^60^ This effect is particularly important for thick perovskite films required in X-ray absorption applications.

Overall, device-level heterostructure and interface engineering provide powerful means to simultaneously optimize charge extraction, suppress leakage current, stabilize internal electric fields, and mitigate ion migration. These strategies are therefore central to achieving high-performance perovskite X-ray detectors with low detection limits, high operational stability, and compatibility with practical imaging systems.

## Challenges and perspectives

Halide perovskites have rapidly emerged as promising materials for next-generation X-ray detectors because of their strong X-ray attenuation, high mobility-lifetime product, low-cost processing, and compatibility with both direct and indirect detection modes. More importantly, the versatile chemistry and defect-tolerant nature of halide perovskites provide broad opportunities for heterostructure engineering, enabling precise regulation of charge transport, interfacial energetics, ion migration, and carrier recombination. More recently, heterostructure engineering has provided an effective route to further enhance detector sensitivity, suppress dark current, improve carrier extraction, and stabilize device operation. Despite the remarkable progress, several key challenges still limit the transition of perovskite X-ray detectors from laboratory demonstrations to practical imaging systems, particularly for high-resolution and low-dose medical imaging. In this regard, heterostructure engineering, coordinated with materials design, interface engineering, device architecture, and system integration is expected to play a central role in overcoming the current bottlenecks and accelerating device commercialization.

**Figure 7:**
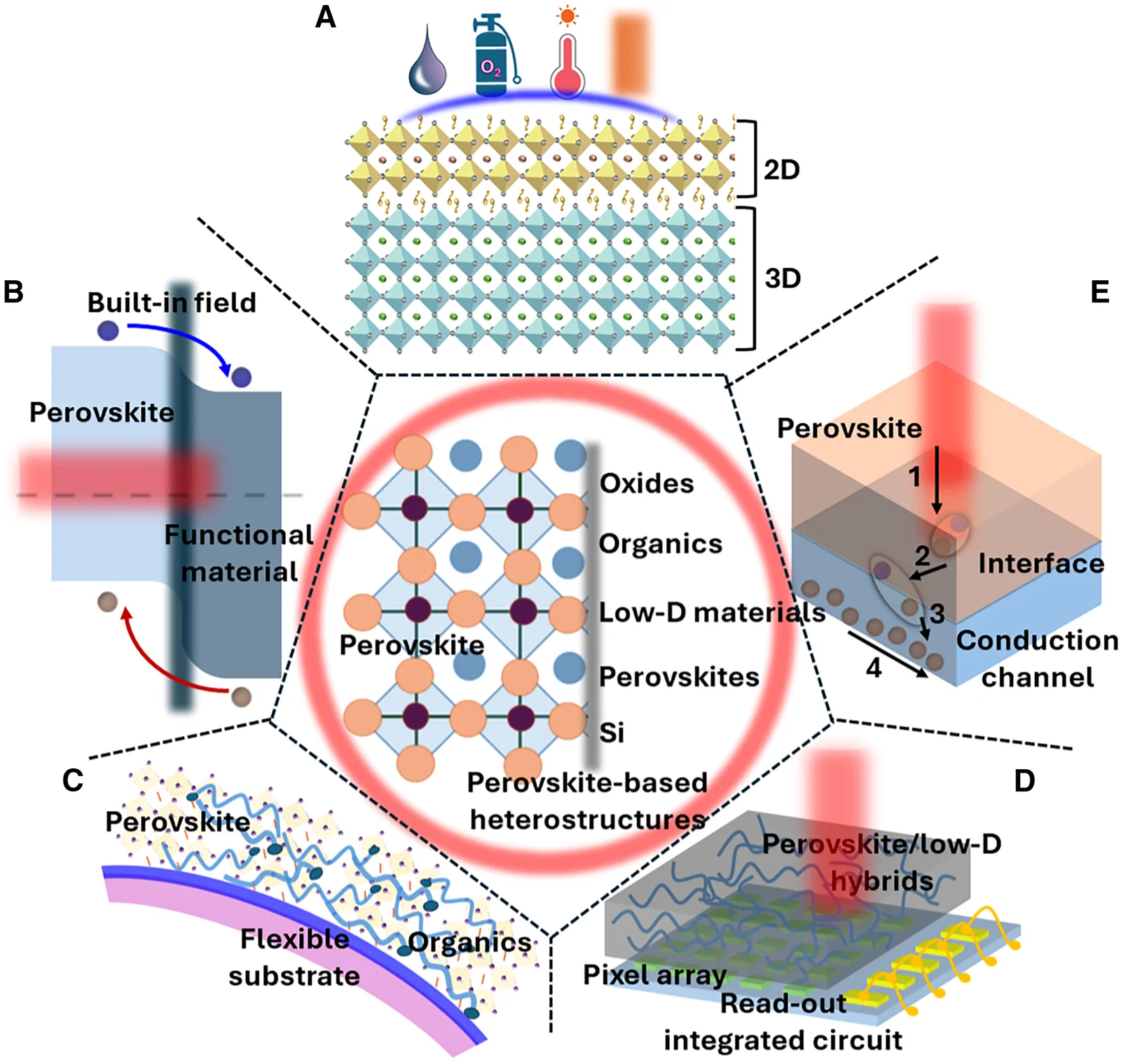
Future perspectives of perovskite-based heterostructure X-ray detectors (A) Improving device stability: 2D/3D mixed-D perovskite heterostructures effectively enhance environmental stability while preserving efficient charge extraction.^49^ (B) Enhancing charge transfer: type II heterojunctions facilitate the radiation-excited carrier separation and promote directional carrier extraction. (C) Realizing flexible X-ray detection devices: perovskite/polymer composite heterostructures improve mechanical flexibility. (D) Promoting device integration: scalable growth of perovskite-based heterostructures on pixelated substrates could improve operational uniformity. (E) Improving imaging performance: heterostructure engineering facilitates efficient charge carrier generation and leads to ultrahigh sensitivity. Reproduced with permission (A).

A primary challenge is the insufficient operational stability of halide perovskites under moisture, thermal stress, electrical bias, and continuous X-ray irradiation. Hybrid perovskites containing organic cations are vulnerable to decomposition and ion migration, whereas all-inorganic perovskites may still suffer from phase instability. These issues become more severe under high electric fields and prolonged irradiation conditions typically required for X-ray detection. Heterostructure engineering offers an effective route to address these limitations. Constructing low-dimensional/3D heterostructures, graded junctions, or passivated interfaces can suppress defect formation and increase ion migration barriers.^49^^,^^61^^,^^62^ In particular, 2D/3D perovskite heterostructures have demonstrated improved environmental robustness and reduced ionic conductivity while preserving efficient charge extraction (Figure 7A).^49^ Similarly, integrating wide-bandgap semiconductors or insulating interlayers can block undesired carrier injection and suppress dark current. Future studies should focus on understanding interfacial ion dynamics and radiation-induced defect evolution in heterostructures under realistic operating conditions.

Another major issue is the trade-off between high sensitivity and low noise. High electric fields are generally required to improve charge collection efficiency and sensitivity, but they simultaneously increase dark current and baseline drift. Interfacial heterostructures provide opportunities to decouple these competing effects. Rational band alignment between perovskites and transport layers can establish built-in electric fields that facilitate charge separation without relying solely on large external bias.^5^^,^^10^ In particular, type II heterojunctions are particularly attractive because they facilitate the radiation-excited carrier separation, promote directional carrier extraction, and suppress interfacial recombination (Figure 7B). In addition, Schottky contacts, blocking layers, and van der Waals heterostructures with 2D materials can effectively reduce carrier injection and leakage current, thereby improving SNR and lowering detection limits. Developing heterostructures with optimized energy-level alignment and minimized interfacial traps will therefore remain a key research direction.

The fabrication of thick, large-area, and flexible detector layers also remains challenging. Efficient X-ray absorption typically requires active layers with thicknesses ranging from hundreds of micrometers to millimeters, which are difficult to achieve uniformly using conventional solution-processing methods. Thick perovskite wafers or polycrystalline films often contain pinholes, grain boundaries, and residual strain, all of which deteriorate carrier transport and operational stability.^16^ Heterostructure engineering can provide effective solutions by combining different dimensional or compositional perovskites to simultaneously optimize absorption, transport, and mechanical robustness.^63^ For example, vertically graded heterostructures may enable efficient carrier extraction across thick films, while polymer/perovskite composite heterostructures could improve mechanical flexibility and environmental tolerance (Figure 7C). Flexible X-ray detectors based on embedded or laminated heterostructures are especially promising for wearable imaging systems and curved-surface detection.

Beyond materials and device design, integration with readout electronics and detector arrays is essential for real-world imaging systems. Most current demonstrations remain limited to single-pixel devices, whereas real imaging systems require large-scale detector arrays integrated with thin-film transistor backplanes and readout circuits.^1^ However, conventional microfabrication processes used for commercial X-ray detectors are often incompatible with the chemical sensitivity and thermal instability of halide perovskites. Heterostructure engineering may facilitate device integration by improving interfacial compatibility and reducing defect density at the contact regions. Direct growth of perovskite heterostructures on charge-transport layers or pixelated substrates could minimize interfacial resistance and improve operational uniformity (Figure 7D). Furthermore, scalable fabrication methods for multilayer heterostructures, including blade coating, slot-die coating, and lamination techniques, should be further explored for industrial manufacturing.

Improving imaging performance, particularly spatial resolution and imaging fidelity, is equally critical for medical applications. Current perovskite imaging systems still lag behind commercial technologies in terms of high-resolution imaging under clinically relevant conditions. Heterostructure engineering can help address this challenge by enabling carrier-density modulation and efficient charge carrier generation, resulting in the low noise current, high photoconductive gain, and thus ultrahigh sensitivities (Figure 4E).^64^^,^^65^ Carefully designed interfacial layers can suppress charge spreading and improve MTF, thereby enhancing image sharpness. Moreover, scintillator heterostructures combining perovskites with luminescent or photonic materials may further improve light extraction efficiency and reduce optical crosstalk in indirect detectors.

Finally, future progress should move beyond record sensitivity values toward application-oriented benchmarking. Translating perovskite X-ray detectors from laboratory prototypes to clinical and industrial applications requires standardized evaluation protocols and long-term reliability testing. Future research should move beyond reporting peak sensitivity values and instead focus on operational durability, reproducibility, imaging quality, and system-level integration.^1^^,^^66^ In this context, interdisciplinary collaboration among materials scientists, device physicists, electronics engineers, and medical researchers will be essential.

Looking forward, heterostructure engineering is expected to become one of the most powerful strategies for advancing halide perovskite X-ray detectors. By tailoring interfacial energetics, suppressing ion migration, regulating carrier transport, and improving device integration, heterostructures can simultaneously address multiple limitations of current perovskite systems. Continued progress in interface design, scalable fabrication, and mechanistic understanding will likely enable perovskite X-ray detectors to evolve from proof-of-concept devices toward practical imaging technologies for medical diagnostics, security inspection, and scientific instrumentation.

## Acknowledgments

This work was financially supported by the Discovery Early Career Researcher Award (DECRA) scheme (DE180100167) from the 10.13039/501100000923Australian Research Council (ARC).

## Author contributions

F.L. contributed to the conceptualization, literature review, visualization, writing – original draft, writing – review and editing, and funding acquisition.

## Declaration of interests

The authors declare no competing interests.
